# Textural Effects on Perceived Satiation and Ad Libitum Intake of Potato Chips in Males and Females

**DOI:** 10.3390/foods9010085

**Published:** 2020-01-13

**Authors:** Jimmy Cahayadi, Sze Ying Leong, Indrawati Oey, Mei Peng

**Affiliations:** 1 Department of Food Science, University of Otago, P.O. Box 56, Dunedin 9054, New Zealand; jimmy.cahayadi@otago.ac.nz (J.C.); sze.leong@otago.ac.nz (S.Y.L.); indrawati.oey@otago.ac.nz (I.O.); 2 Riddet Institute, Private Bag 11 222, Palmerston North 4442, New Zealand

**Keywords:** food texture, satiation, food intake, gender differences, snack eating behaviour

## Abstract

Food texture plays a critical role in influencing an individual’s perceived satiation and ad libitum intake. It remains unclear, however, whether such textural changes can also affect snack consumption. This study aimed to address this question by testing for changes in perceived satiation and ad libitum intake of two types of potato chips with varying hardness. In addition, the observed effect was compared across gender groups. With a crossover design, 74 participants (31 females and 43 males) performed a food consumption task for two types of chips produced from potatoes that were either untreated or treated with pulsed electric fields (PEF) technology. Sensory analyses indicated that these two types of chips had comparable hedonic value, despite a clear textural difference. Across sexes, the results revealed a significant difference in perceived satiation for the two types of chips (*p* = 0.009), but not in intake. By contrast, analyses of males alone revealed that male participants rated PEF-treated chips to be more satiating than the control chips and correspondingly consumed less (*p* < 0.05). Overall, findings from the study suggest that modifications of food texture can be a helpful tool in reducing energy intake from snack consumption. The contrasting results from different gender groups highlight the importance of considering gender effects in studies of eating behaviour.

## 1. Introduction

Obesity is currently a worldwide health problem [1]. Although obesity is caused by myriad factors, overconsumption is one of the key contributors to this epidemic [2]. In the current food environment, excessive intake occurs for both main meals and also for snack foods—the latter defined as food consumed between meals. Indeed, research has shown that snacking alone can constitute up to a third of an individual’s total energy intake [3,4]. This statistics has spurred researchers to specifically investigate factors that can influence snack intake [5]. In the current field of food technology, it becomes increasingly important to identify factors that help to reduce people’s caloric intake while maintaining hedonic appeal of food products.

Perceived satiation refers to the feeling of fullness that leads to meal termination [6]. Perceived satiation plays an important role in determining food intake during premeal planning and meal consumption [6,7]. It can be measured by quantifying either self-reported satiation or ad libitum intake. A range of external and internal factors can affect an individual’s satiation [6]. In addition, research has increasingly recognised that sensory experiences associated with food consumption, such as taste and smell, can also play an important role in satiation. Previous studies, for instance, have shown that foods with strong tastes or smells can lead to higher satiation [8,9]. More recently, food texture is identified to be another important factor that influences satiation when consuming liquid or semi-liquid foods. For example, Mars et al. [10] demonstrated that viscous yoghurt was perceived as more satiating than its thinner counterpart. Furthermore, Zijlstra et al. [11] showed similar results when comparing the satiation of chocolate milk versus chocolate custard, notwithstanding the intake being equal. A number of studies have found food texture influences perceived satiation via changes in oral processing duration—with prolonged oral processing assumed to generate increased release of aromatic and gustatory signals [11,12].

Textural effects on food consumption have also been observed in solid foods. Larsen et al. [13] tested effects of texture complexity on perceived satiation and intake of gel-agar disks and found that the high-complex texture led to increased perceived satiation as opposed to the low-complex texture. Similarly, another study using food gels also found harder texture reduces food consumption by 21% [14]. Furthermore, Forde et al. [15] found that participants had higher consumption when they were given mashed food (soft texture) as opposed to unmashed food (hard texture). Bolhuis et al. [16] compared the intake of hamburger (i.e., hard vs. soft) and rice salads (i.e., rice vs. risotto), and found that participants reported similar satiation level but had about 13% more consumption with the soft version. These findings consistently suggest that hard food texture can lead to reduction in energy intake.

Recent studies have also investigated the effect of texture on food intake with relatively subtle modifications [17,18,19]. For instance, Mosca et al. [18] compared the intake of two types of yoghurt that differed in viscosity by a factor of 1.57 and 1.81. They found that this subtle change could reduce the consumption by 5% but did not affect the satiation rating. Similarly, by adding fruit cubes to increase the viscosity of yoghurt by 2.6 fold, Tarrega et al. [17] found an increase in the perceived satiation by 23% in low viscosity yoghurt and 6% in thick viscosity yoghurt. While the effect of subtle textural modification on food intake appeared to be consistent in semi-solid foods, Zijlstra et al. [19] did not observe such effect in solid food. They tested three types of solid foods (i.e., candy, luncheon meat, and meat replacer), with varying levels of hardness and they did not find any significant difference in the ad libitum intake. It thus remains unclear whether more subtle textural differences can also lead to changes in eating behavior. Moreover, these inconsistent findings indicate a need for more research with a broader range of food models.

Recent studies in the area of eating behaviour have indicated the importance of considering gender specific differences [20,21]. Indeed, males and females differ in their sensory perceptions [22], as well as brain responses to food [23], and cognitive processes in making dietary decisions [24]. Specifically, with regards to textural effects, McCrickerd et al. [25] detected intriguing evidence for possible gender differences. Specifically, females responded to textural differences by adjusting the intake of fruit juice, whereas males showed no such sensitivity. Recently, studies also observed gender differences in solid food [26,27]. Both studies observed that males had a larger bite size and faster eating rate compared to females when consuming solid foods. These recent findings suggest that gender difference in food textural effects should be further considered.

The present study aims to (1) test the effects of food texture on ad libitum intake and perceived satiation of common snack food (potato chips) and (2) examine the effects of gender on responses to textural change. We employ a food processing technology—pulsed electric fields (PEF)—to manipulate food texture with minimal or no impact on any additional sensory factors. By applying high voltage short electric pulses to the food, this technology alters the structural integrity of the plant tissues and evidently modifies the texture of solid and liquid foods [28]. Typically, foods treated with PEF exhibit harder (i.e., ‘crunchier’) texture than untreated foods [29]. We hypothesized that the PEF-treated (harder texture) chips are associated with increased perceived satiation and reduced ad libitum intake. Findings from this study will provide new insights into the effects of food texture on energy intake from consuming snacks. These results are also of interest to food technologists with regards to novel use of PEF technology. In addition, we hypothesised that males and females may exhibit differential responses to such textural variation.

## 2. Materials and Methods

### 2.1. Participants

Participants were recruited from the general population of Dunedin, New Zealand, using flyers and social media. All participants were healthy, between the age of 18–50 years, not being on a diet program, and frequent consumer of potato chips (i.e., once a week). Notably, a large proportion of the participants were university students or staff. At the point of screening, participants were also asked to complete a Dutch Eating Behaviour Questionnaire (DEBQ) [30].

A total of 74 individuals (Female = 31) participated in the study. Table 1 summarizes the participants’ characteristics. A power analysis was performed based on our previous data on self-reported satiation scores. The analysis indicated a suitable sample size of 70 with β = 0.2 and α = 0.95. We recruited a total of 77 participants with a 10% attribution rate. Of those, 74 participants completed this study. All participants gave written consent and received a monetary reimbursement at the end of the study. The University of Otago Human Participation Ethics Committee approved the study (Ref: 18/043).

### 2.2. Food Samples Preparation

The potato chips used in this study were manufactured at our food-grade processing pilot plant. Potatoes were sourced from a local orchard (Agria cultivar; harvested on 7 May 2018 in Invercargill, Invercargill, New Zealand) and stored in a controlled environment (10–15 °C) until being processed. Only undamaged tubers with similar weight, size, and dimensions were selected for making the chips.

Production of potato chips comprised of five main steps: Potato pretreatment either with or without the pulsed electric fields (PEF) technology, slicing, blanching, drying, and frying. To minimize biological and postharvest differences between the tubers, a paired sample design was used in this study. Whole potatoes were picked randomly from the storage on the production day, then peeled by hand, and cut in half. The first half of the tuber was treated with PEF processing (hereafter referred to as “PEF-treated” sample), while the other half tuber was used as the control (i.e., samples without PEF treatment). PEF processing was conducted in a batch configuration using a pilot-scale PEF system (ELCRACK^®^ HVP5, DIL, Quackenbrueck, Germany), equipped with a digital storage oscilloscope (Model UTD2042C, UNI-Trend Group Limited, Dongguan, China) to monitor the shape of the delivered electric pulses.

Both PEF-treated and control potatoes were immediately sliced into 1.5 mm thick slice using an in house manufactured mechanical slicer. Potato slices were then blanched in boiling water for approximately 2.5 min. Subsequently, potato slices were fried in canola oil (Sunfield, Invercargill, Otago, New Zealand) at 180 ± 5 °C for under 3.5 min using a deep fryer (Blue Seal electric fryer Evolution series E44E—450 mm, Birmingham, UK). Potato chips were cooled on a metal mesh racks for at least 5 min and to allow any excess of oil to drip. A 1% (*w*/*w*) food grade sodium chloride was added to the potato chips as a flavouring. At last, 75 g (±5 g) of potato chips were packed in a vacuum packaging and stored in a cool dry environment.

### 2.3. Physicochemical Measures of Food Samples

Samples of potato chips produced on three different production days were selected for testing fat content (i.e., Soxhlet extraction) and water activity (using a benchtop water activity meter, Aqualab 4TE model, FF Instrumentation, Christchurch, New Zealand). The fat content was then used to calculate the energy density of the potato chips.

Additionally, twelve potato crisps were selected randomly from each bag for texture analysis. A penetration test was conducted on individual chips using a texture analyzer (TA. HDplus, Stable Micro Systems Limited, Surrey, UK) fitted with a stainless steel spherical probe (Part P/5S, 5 mm diameter) and a 5 kg load cell. Each chip was centrally placed on rectangular support attached to the heavy duty platform. The probe was set at 20 mm above the chips, and the penetration test was performed as follows: Pre-test speed of 1 mm/s and a test speed of 0.5 mm/s. The maximum force for the highest peak (force vs. distance) represents the force required to penetrate through the potato crisp until fracture occurs.

### 2.4. Experimental Procedures

The eating behaviour study adopted a crossover design. Each participant attended two study sessions with one week apart. In each session, participants were asked to consume either the PEF-treated or the control potato chips ad libitum. The order of tests was counterbalanced across the participants. The experiment was carried out by cohorts, with a maximum of six participants in a cohort. Notably, the experiment was conducted in the morning between 9 and 12 h. Such experimental time was chosen because it allowed the constraint of participants’ hunger levels, although it was not a typical time for snack consumption.

Upon arrival, participants were first asked to report their hunger level on a 150 mm visual analogue scale (VAS) [31]. Afterwards, participants were served with a breakfast meal (i.e., oat porridge) and asked to consume ad libitum until they felt comfortably full. After consumption, participants rated their hunger level again on a VAS.

At 45 min after breakfast, the participants were led into a standard sensory booth and given a bowl of potato chips for their consumption while watching a 30 min documentary video clip. The content of the video clip was not food related. During the test, the participants were told that they could ask for refills at any time. At the end of the video, each participant’s intake was calculated based on left over chips. Subsequently, participants were asked to rate their fullness (“not full at all to extremely full”), liking for the chips (“extremely dislike” to “extremely like”), crispiness (“not crispy at all” to “extremely crispy”), and crunchiness of potato chips (“not crunchy at all” to “extremely crunchy”) on a VAS scale. All questionnaires were developed and distributed by Qualtrics software. At the end of the study, the participant’s height and weight were measured.

### 2.5. Statistical Analyses

A series of *t*-tests were used to examine differences between the two types of potato chips in terms of their physicochemical (i.e., water activity and rheology measures) and the sensory (i.e., liking, crunchiness, and crispiness) properties. Independent sample t-tests were employed to assess whether there was a significant difference between the male and female sample groups in terms of age, BMI, and DEBQ scores. Separate mixed model repeated measures ANOVA were then conducted to analyze differences in ad libitum intake and perceived satiation for these two types of chips. In this model, the within subject variable was defined as the food samples (i.e., PEF-treated and control potato chips), and the between-subject variable was defined as gender. Post-hoc tests, based on simple effects tests, with Bonferroni correction were used to explain any significant effects. An alpha level of 5% was used for detecting significant differences. These analyses were performed with SPSS Statistics (IBM Corporation, v25, New York City, NY, USA).

## 3. Results

### 3.1. Physiochemical Parameters of the Potato Chip Samples

**Figure 1:**
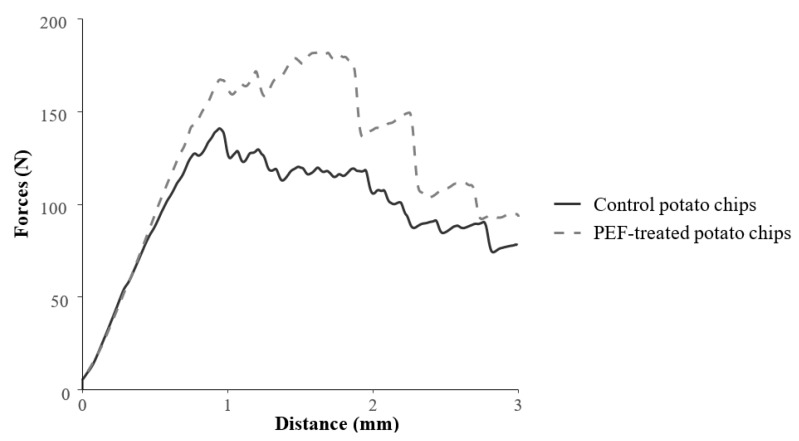
Plot of penetrating forces (N) against distance (mm) for the two testing potato chips measured by the textural analyser.

Table 2 summarizes the physicochemical parameters of potato chips used in this study. On average, the energy density was 2.97 ± 0.20 kcal/g for the PEF-treated potato chips and 3.22 ± 0.22 kcal/g for the control potato chips. The texture analysis recorded that the maximum forces required to penetrate PEF potato chips was 182.26 N and for control chips was 140.91 N. The t-test on the data from the texture analysis showed that on average, the PEF-treated potato chips required significantly (*p* < 0.01) stronger force to penetrate through the potato chip compared to the control chips (see Figure 1), suggesting the former had a harder texture.

### 3.2. The Perceptual Difference in the Food Textures

The results from the *t*-tests on sensory data indicated that participants rated the PEF-treated potato chips to be significantly crunchier than the control chips (*p* < 0.01). Nevertheless, liking score and crispiness rating were statistically comparable for the two chips (Table 2).

### 3.3. Effect of Texture Differences on Ad Libitum Intake

**Figure 2:**
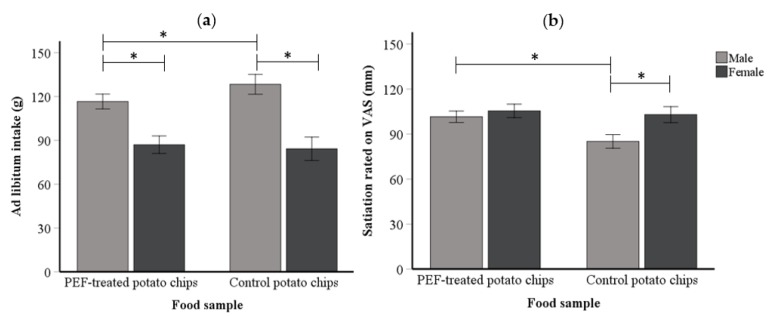
Comparison between genders on their ad libitum intake (**a**) and perceived satiation (**b**) of the PEF and control potato chips. The PEF-treated potato chip (associated with harder texture) refers to the potato chips treated with the pulsed electric fields (PEF) technology and the control potato chips refer to untreated with the PEF potato chips. The * indicates significant difference at *p* ≤ 0.05.

A series of independent sample t-tests were employed to assess differences between the male and female gender group in terms of age, BMI, and DEBQ scores. The results suggested no statistical difference between these two groups. These variables were therefore not included in the following analyses of satiation and intake data. The mixed model ANOVA showed that food texture did not have a significant main effect on ad libitum intake (in gram; g). On average, the participants consumed a similar amount of the PEF-treated (M = 104.20 g, SD = 36.31) and the control potato chips (M = 109.86 g, SD = 49.47). Further analysis incorporating genders and food samples (PEF-treated and control potato chips) showed that male participants consumed significantly less of the PEF-treated potato chips (M = 116.59 g, SD = 30.42) than the control potato chips (M = 128.34 g, SD = 50.53, *p* = 0.03; see Figure 2). By contrast, female participants consumed a similar amount of both potato chips (PEF-treated potato chips: M = 87.01 g, SD = 37.25 and control potato chips: M = 84.21 g, SD = 34.81, see Figure 2).

### 3.4. Effect of Texture Differences on Perceived Satiation

By contrast, the mixed model ANOVA of the satiation data showed a highly significant main effect of the food sample (F (1,72) = 7.25, *p* = 0.009, η^2^ = 0.092). Specifically, participants rated the PEF-treated potato chips to be significantly more satiating (M = 103.14, SD = 24.85) than the control potato chips (M = 92.57, SD = 30.80). Moreover, food samples and genders were found to be significantly interacted with each other in influencing the perceived satiation (F (1,72) = 3.94, *p* = 0.05, η^2^ = 0.052). The post-hoc test with Bonferroni corrections revealed that male participants rated the PEF-treated potato chips significantly more satiating (M = 101.49, SD = 26.84) than the control potato chips (M = 85.09, SD = 34.34, *p* = 0.001; see Figure 2). However, the female participants gave similar responses across the two food samples.

## 4. Discussion

Our study examines the effects of snack food texture on perceived satiation and ad libitum intake, and evaluates these effects against gender. Based on data pooled across genders, textural differences in snack food were demonstrated to alter an individual’s perceived satiation, but not their energy intake. However, additional gender specific analyses suggest that only male participants report increased satiation and show reduced consumption in response to ‘crunchy’ texture (i.e., PEF-treated potato chips). By contrast, female participants apparently exhibit similar sensitivities regardless of texture.

Recent studies have shown that subtle texture modifications of semi-solid food could affect food intake [17,18,32]. While Zijlstra et al. [19] did not detect any such effects in solid food, the present study found that subtle changes in food texture can affect intake. These contrasting findings might be partly attributable to differences in eating behaviour between main meals and snacks. For main meals, research has demonstrated that perceived satiation and intake are often integrally linked, with elevated perceived satiation resulting in termination of intake [6]. However, perceived satiation plays a different role in determining snack intake. For example, Chapelot [33], in a review, revealed that snack consumption had little to no effect on self-reported hunger levels, insulin response, or subsequent food intake. The apparent distinctions between snacks and main meals may be driven by their different mechanisms. Indeed, consumption of main meals, in most cases, is to satisfy homeostatic needs, whereas consuming snacks is driven by hedonic demands [34]. Findings from the present study show that textural differences of snack foods might alter an individual’s perceived satiation, but not necessarily change the eventual energy intake. These novel observations highlight the need for more research that specifically investigates snack consumption.

An additional explanation for the contrasting findings between the present and past studies may involve the experimental protocol. In the present study, participants were asked to consume snacks in the presence of a distractor. This protocol was adopted to improve the ecological validity of the study, although it poses an additional challenge when comparing current and previous findings. A study from Wansink and Kim [35] have shown that, in an eating scenario with distractions, individuals pay less attention to sensory cues, e.g., food texture. Hypothetically, diminished attention to food sensory cues can lead to variation in perceived satiation and food intake. More research is required to confirm the additional effects of such distraction.

Separate analyses of data based on gender suggest a substantial difference between sexes regarding changes in texture. Such gender effects have been reported in just a single previous study. Specifically, McCrickerd et al. [25] noted that only females responded to textural differences in fruit juice (i.e., reduced intake for drinks with thicker consistency). By contrast, the present study found that only male participants showed varied responses based on food texture, whereas female participants showed no such changes based on textural differences. Some of these discrepant findings from the current and previous studies may be partly attributable to the testing food model (i.e., solid and liquid food). Liquid foods, for instance, can be consumed quickly [36], whereas solid foods require longer oral processing and may thus show different gender-based differences. A previous study also noted gender-based differences in intake rates for solid foods [37], which could perhaps contribute to the observed patterns between sexes.

A caveat of the current study is that a single snack product was tested. Caution should apply when generalizing these findings to other foods because variations in sensory properties, such as flavour, are expected to have differential effects on perceived satiation and intake [9]. In addition, the present study was conducted in the morning, which is not a typical time for snack consumption.

## 5. Conclusions

Increasing snack consumption is closely related to the current obesity epidemic. In the current field of food technology, it becomes increasingly important to identify factors that help to reduce people’s caloric intake while maintaining the hedonic appeal of food products. The present study examined the effects of textural changes on perceived satiation and intake of snack food, and obtained evidence for textural effects in males. Findings from the study suggest that modifications of food texture can be a helpful tool in reducing energy intake from snack consumption. Moreover, the present findings indicate variation in eating behaviour between snack and meal consumption. These findings also reiterate the importance of considering gender difference in studies of effects of oral processing on food intake.

## Figures and Tables

**Table 1:** Summary of participants’ characteristics.

	Females (*n* = 31) M (SD)	Males (*n* = 43) M (SD)
Age	24.1 (2.7)	26.2 (3.4)
Body-mass-index	23.4 (3.4)	25.7 (3.9)
DEBQ-total	2.5 (0.9)	2.1 (0.8)
DEBQ-restraint	2.6 (1.1)	2.5 (0.9)
DEBQ-emotional	2.7 (1.3)	2.2 (1.2)
DEBQ-external	3.8 (0.7)	3.6 (0.9)

**Table 2:** Summary of means (and standard deviation) of the physicochemical and sensory parameters of the PEF-treated and control potato chips used in this study.

		PEF-Treated Sample	Control Sample	*t*-Test	
				*t*-Value	*p*-Value
Physicochemical parameters	Texture measurement ^1^	124.81 ± 46.05	97.24 ± 29.98	−51.23	<0.01
	Water activity (aw at 25 °C)	0.35 ± 0.00	0.35 ± 0.02	0.29	0.79
Sensory parameters	Liking	84.81 ± 30.08	80.22 ± 30.40	1.53	0.13
	Crunchiness	97.45 ± 28.90	85.99 ± 27.36	3.25	<0.01
	Crispiness	91.07 ± 27.21	91.74 ± 28.67	−0.17	0.87

^1^ Mean forces (N) required to break potato chips into halves.

## Supplementary Materials

Supplementary File 1
